# Deficiency of D‐alanyl‐D‐alanine ligase A attenuated cell division and greatly altered the proteome of *Mycobacterium smegmatis*

**DOI:** 10.1002/mbo3.819

**Published:** 2019-03-03

**Authors:** Yingfei Chen, Yuefei Xu, Shufeng Yang, Sheng Li, Wenyong Ding, Wenli Zhang

**Affiliations:** ^1^ Dalian Yuming Senior High School Liaoning China; ^2^ Biochemistry and Molecular Biology Department of College of Basic Medical Sciences Dalian Medical University Dalian China; ^3^ Department of Microbiology Dalian Medical University Dalian China

**Keywords:** cell division, D‐alanyl‐D‐alanine ligase A (DdlA), DdlA deficiency, *Mycobacterium smegmatis*, proteome

## Abstract

D‐Alanyl‐D‐alanine ligase A (DdlA) catalyses the dimerization of two D‐alanines yielding D‐alanyl‐D‐alanine required for mycobacterial peptidoglycan biosynthesis, and is a promising antimycobacterial drug target. To better understand the roles of DdlA in mycobacteria in vivo, we established a cell model in which DdlA expression was specifically downregulated by *ddlA* antisense RNA by introducing a 380 bp *ddlA* fragment into pMind followed by transforming the construct into nonpathogenic *Mycobacterium smegmatis*. The *M. smegmatis* cell model was verified by plotting the growth inhibition curves and quantifying endogenous DdlA expression using a polyclonal anti‐DdlA antibody produced from the expressed DdlA. Scanning electron microscopy and transmission electron microscopy were used to investigate mycobacterial morphology. Bidimensional gel electrophoresis and mass spectrometry were used to analyze differentially expressed proteins. Consequently, the successful construction of the *M. smegmatis* cell model was verified. The morphological investigation of the model indicated that DdlA deficiency led to an increased number of Z rings and a rearrangement of intracellular content, including a clear nucleoid and visible filamentous DNA. Proteomic techniques identified six upregulated and 14 downregulated proteins that interacted with each other to permit cell survival by forming a regulatory network under DdlA deficiency. Finally, our data revealed that DdlA deficiency inhibited cell division in mycobacteria and attenuated the process of carbohydrate catabolism and the pathway of fatty acid anabolism, while maintaining active protein degradation and synthesis. N‐Nitrosodimethylamine (NDMA)‐dependent methanol dehydrogenase (MSMEG_6242) and fumonisin (MSMEG_1419) were identified as potential antimycobacterial drug targets.

## 1 INTRODUCTION

Tuberculosis (TB), an infectious disease caused by *Mycobacterium tuberculosis*, remains a major threat to human health in terms of worldwide morbidity and mortality (World Health Organization, 2018). Currently, the number of TB deaths and new TB cases are continuously increasing each year, and the new TB epidemic was assessed in the 2018 Global Tuberculosis Report (World Health Organization, 2018). The report indicated an estimated 10.0 million new TB cases and approximately 1.3 million TB deaths occurred worldwide in 2017 and that the global treatment success rate remained low, at 55%, because current therapies are ineffective for cases with multiple drug‐resistant (MDR) *M. tuberculosis* infection (MDR‐TB). An estimated 18% of previously treated TB cases and 3.5% of new TB cases worldwide are MDR‐TB (World Health Organization, 2018). Novel anti‐TB compounds are not being developed quickly enough to keep pace with the rapid spread of MDR*‐M. tuberculosis* (Ba Diallo et al., 2017). Thus, there is an urgent requirement to develop novel anti‐TB drug targets or anti‐TB drugs to fight against MDR‐*M. tuberculosis*.

D‐Alanyl‐D‐alanine ligase A (DdlA) is an attractive potential anti‐TB drug target (Yang et al., 2018). DdlA catalyses the ATP‐dependent dimerization of two D‐alanine molecules to yield D‐alanyl‐D‐alanine, which is required for the biosynthesis of peptidoglycan (PG); PG is essential for preserving cell integrity by preserving internal osmotic pressure and maintaining the defined cell morphology and cell division (Bruning, Murillo, Chacon, Barletta, & Sacchettini, 2011; Kieser et al., 2015; Yang et al., 2018). Theoretically, the biosynthesis of PG is an ideal target for anti‐TB drug design because the complete pathways of PG biosynthesis are not present in mammalian cells. PG is composed of linear glycan chains consisting of repeating disaccharide units consisting of β‐1,4‐linked N‐acetylglucosamine‐N‐acetylmuramic acid moieties and crosslinked tetrapeptide chains consisting of an L‐alanyl‐D‐isoglutaminyl‐meso‐diaminopimelyl‐D‐alanine moiety that is bound to N‐acetylated muramic acid. Crosslinks between two adjacent oligopeptide chains are formed by the binding of meso‐diaminopimelate tetrapeptide side chains to a D‐alanine residue on the adjacent tetrapeptide, in turn releasing the terminal D‐alanine residue (Barreteau et al., 2008; Macheboeuf, Contreras‐Martel, Job, Dideberg, & Dessen, 2006; Yang et al., 2018). The crosslinked PG confers tensile strength to the bacterial cell wall. Thus, the inhibition of PG crosslinking causes extensive cell wall weakening or cell death; DdlA is involved in PG crosslinking by supplying the indispensable D‐alanyl‐D‐alanine. In addition, compounds with structural similarity to D‐alanine can competitively inhibit DdlA activity with fewer side effects, because D‐alanine is absent in humans. Therefore, in combination with the essentiality of the *ddlA* gene in *M. tuberculosis*, the characteristics of DdlA make it an ideal target for anti‐TB drugs.

In fact, the particularly interesting nature of DdlA as a novel anti‐TB drug target that has been indicated by the clinical application of D‐cycloserine as an effective second‐line antibiotic against *M. tuberculosis* (Halouska et al., 2014; McCoy & Maurelli, 2005; Prosser & de Carvalho, 2013). However, the use of D‐cycloserine, which targets DdlA and alanine racemase as structural analogues of D‐alanine, is restricted in TB treatment because of its serious neurological side effects (Yang et al., 2018). D‐Cycloserine overdose has been reported to cause central nervous system side effects such as drowsiness, headaches, vertigo, depression, paraesthesias, dizziness, dysarthria, confusion, hyperirritability, convulsions, psychosis, tremors, paresis, seizures, and coma by activating the N‐methyl‐D‐aspartate‐type glutamate receptor (Chen, Uplekar, Gordon, & Cole, 2012; Halouska et al., 2014; Schade & Paulus, 2016). To design a nontoxic compound that targets DdlA and to better understand the roles of DdlA in mycobacteria in vivo, an extensive study on DdlA was performed using a *Mycobacterium smegmatis* model of DdlA downregulation induced by *Sm‐ddlA* antisense RNA.

## 2 MATERIALS AND METHODS

### 2.1 Strains and plasmids

The properties of the plasmids and bacterial strains used in this study are shown in Table 1. *Escherichia coli* NovaBlue and BL21 (DE3) cells are routinely grown on Luria‐Bertani (LB) agar or in LB broth at 37°C. *M. smegmatis* mc^2^155 cells are routinely grown on LB agar or in LB broth supplemented with 0.05% Tween 80 at 37°C. The final antibiotic concentrations used were as follows: 100 μg/ml ampicillin (Amp) and 50 μg/ml kanamycin for *E. coli* and 25 μg/ml kanamycin for *M. smegmatis*.

**Table 1:** Bacterial strains and plasmids used in this study

Strains/plasmids	Description	Source
Strains		
*Escherichia coli* NovaBlue	Used for cloning and plasmid propagation	Novagen
*E. coli* BL21 (DE3)	Used for expressing the *Sm*‐DdlA protein	Invitrogen
*Mycobacterium smegmatis* mc^2^155	Wild‐type; nonpathogenic; a surrogate model in lieu of the pathogenic and slower‐growing *Mycobacterium tuberculosis*; used as a DNA template to amplify *M. smegmatis ddlA* and *Sm‐ddlA* antisense RNA	ATCC
pMind‐*Sm‐ddlA*‐AS in *M. smegmatis*	*M. smegmatis* cell model; *M. smegmatis* mc^2^155 carrying pMind‐*Sm‐ddlA*‐AS	This study
pMind in *M. smegmatis*	Control *M. smegmatis* cells, *M. smegmatis* mc^2^155 carrying pMind	This study
Plasmids		
pMD18‐T	Carries the *ampR* gene; used for cloning PCR products with adenosine overhanging at the 3’ ends	Takara
pCold II	Carries the *ampR* gene; contains a cold‐inducible promoter and a hexahistidine tag at its N‐terminus; used for expressing the *Sm‐*DdlA protein	Takara
pMD18‐*Sm‐ddlA*	Carries the *ampR* gene; *M. smegmatis Sm‐ddlA* was cloned into the EcoRV site of pMD18‐T	This study
pCold II‐*Sm‐ddlA*	Carries the *ampR* gene; used for cloning and expressing the *Sm*‐DdlA protein in *Escherichia coli*	This study
pMD‐*Sm‐ddlA*‐AS	Carries the *ampR* gene; *M. smegmatis Sm‐ddlA*‐AS was cloned into the EcoRV site of pMD18‐T	This study
pMind	Carries the *kanR* gene; induced by tetracycline; used for the expression of *Sm‐ddlA* antisense mRNA	
pMind‐*Sm‐ddlA*‐AS	Carries the *kanR* gene; the DNA fragment of *Sm‐ddlA* with its upstream region was cloned into the SpeI and NdeI sites of pMind	This study

### 2.2 Gene manipulation and protein expression

*Mycobacterium smegmatis* genomic DNA was used as a PCR template to amplify *Sm‐ddlA* (*MSMEG_2395*), yielding a 1,122 bp DNA fragment. Then, the purified PCR product was cloned into pMD18‐T, generating the pMD18‐*Sm‐ddlA* plasmid. Following sequence confirmation, *Sm‐ddlA* was digested for subsequent ligation into pCold II (Takara, China) to generate pCold II‐*Sm‐ddlA,* which was transformed into *E. coli* BL21(DE3) for DdlA expression. The preparation of cell lysates and the purification and detection of DdlA were conducted as described previously (Yang et al., 2018).

### 2.3 Production of the antibody against *M. smegmatis* DdlA

Purified DdlA was used to immunize mice to produce the anti‐DdlA polyclonal antibody. A 1 mg/ml sample of DdlA (in physiological saline) was emulsified with an equal volume of Freund's incomplete adjuvant (Thermo Scientific) by vortexing vigorously to yield a homogenate of antigen suspension. The prepared antigen suspension was injected subcutaneously into three sites on each female BALB/c mouse (approximately 8 weeks old) on the 1st, 10th, and 17th day. Blood was collected from the orbital venous plexus of the mice on the 24th day. Antiserum was obtained by centrifugation. Then, the specificity of the polyclonal anti‐DdlA antibody was evaluated by Western blot (WB) analysis, and antibody titers were determined by enzyme‐linked immunosorbent assay (ELISA). The colorimetric visualization of the WB was performed using BCIP/NBT solution, and the ELISA was conducted using p‐nitrophenyl phosphate disodium solution.

### 2.4 Establishment of the cell models of *M. smegmatis* DdlA downregulation by *Sm‐ddlA* antisense RNA

The DNA fragment (380 bp) of *Sm‐ddlA‐*AS, including 100 bp upstream of the *Sm‐ddlA* transcription initiation site and 280 bp of the *Sm‐ddlA* sequence at the 5’ end, was amplified from *M. smegmatis* genomic DNA by PCR using the primers *ddlA*‐F, 5’ TAACTAGTGTGACTGCCCCGAACCATC 3’ (the SpeI site is underlined), and *ddlA*‐R, 5’ ATCATATGCTGGATGGTGCCGTCTTCG 3’ (the NdeI site is underlined). The purified PCR fragment was ligated to pMD18‐T to yield pMD‐*Sm‐ddlA*‐AS. Following sequence confirmation, the *Sm‐ddlA*‐AS fragment from pMD‐*Sm‐ddlA*‐AS was cloned into pMind to generate pMind‐*Sm‐ddlA*‐AS (Table 1), whose expression can be induced by tetracycline. The pMind‐*Sm‐ddlA*‐AS was electroporated into competent *M. smegmatis* cells as described previously (Pelicic et al., 1997).

### 2.5 *M. smegmatis* cell growth assays

*M. smegmatis* cells carrying pMind‐*Sm‐ddlA*‐AS were grown in LB broth. Tetracycline was added as an inducer at gradient concentrations of 0 ng/ml, 10 ng/ml, 20 ng/ml, 30 ng/ml, and 50 ng/ml. The growth of *M. smegmatis* cells was determined by monitoring the optical density (OD) of cultures at 600 nm at intervals of 12 hr. The growth curves were plotted as the OD_600 nm_ values versus the incubation time. *M. smegmatis* cells carrying pMind were used as the control. For colony‐forming unit (CFU) analysis, microorganisms grown in LB broth containing 20 ng/ml tetracycline were serially diluted and spread separately on LB agar plates with sterile glass beads at intervals of 12 hr. The colonies on each plate were counted. *M. smegmatis* cells carrying pMind and grown in LB broth containing 20 ng/ml tetracycline were used as the control.

### 2.6 Detection of endogenous DdlA

*Mycobacterium smegmatis* cells carrying pMind‐*Sm‐ddlA*‐AS were grown in LB broth containing tetracycline at concentrations of 20 ng/ml and 0 ng/ml. The cells were harvested after shaking for 36 hr, and cell lysates were extracted as described previously(Yang et al., 2018). Samples of total soluble protein (50 μg) were loaded onto 12% sodium dodecyl sulfate‐polyacrylamide gel electrophoresis (SDS‐PAGE) gels and transferred onto a nitrocellulose (NC) membrane for WB analysis. The transferred membrane was probed by incubating with a polyclonal antibody against DdlA at a 1:2,000 dilution, followed by incubation with an anti‐mouse IgG HRP‐conjugated secondary antibody. The target DdlA bands were visualized using enhanced chemiluminescence (ECL) reagents. *M. smegmatis* cells carrying pMind and grown in LB broth containing 20 ng/ml or 0 ng/ml tetracycline were used as the controls.

### 2.7 Morphological observation

*Mycobacterium smegmatis* cells carrying pMind‐*Sm‐ddlA*‐AS and grown in LB broth containing 20 ng/ml tetracycline were harvested. Then, the harvested cells were fixed in 2.5% glutaraldehyde in 0.1 mmol/ml phosphate buffer (PBS, pH 7.4). Postfixation was performed in 1% osmium tetroxide (OsO_4_) in PBS, followed by dehydration in a graded series of ethanols (20%, 40%, 60%, 70%, 80%, 90%, and 100%). The subsequent transmission electron microscopy (TEM) and scanning electron microscopy (SEM) analyses were conducted as described previously (Zhang et al., 2008).

### 2.8 Protein processing, bidimensional gel electrophoresis and image analysis

Soluble protein was extracted from the harvested *M. smegmatis* cells carrying pMind‐*Sm‐ddlA*‐AS and processed using a clean‐up kit according to the manufacturer's instructions (Bio‐Rad). The protein pellets were dissolved in 50 μl of isoelectric focusing (IEF) buffer (7 mol/L urea, 2 mol/L thiourea, 2% (w/v) CHAPS, and 30 mmol/L Tris, pH 8.0), and the total soluble protein was quantified using a BCA kit. Fifty micrograms of total soluble protein was subjected to bidimensional difference in gel electrophoresis (2D‐DIGE). Proteins were separated in the first dimension according to their pI using Immobiline DryStrips (11 cm, pH 4–7, Bio‐Rad) and in the second dimension according to their molecular weight using 12% SDS‐PAGE gels. The gels were stained using a ProteoSilver Plus SilverStain Kit (Sigma). Image analysis and statistical quantification of relative protein abundances were performed using PDQuest software. Student's *t* test was performed to assess the statistical significance of differentially expressed proteins based on the average spot volume ratio. The protein spots that met the criteria of a fold change (FC) ≥2 or ≤0.5 at a 95% confidence level (Student's *t* test; *p* < 0.05) were selected for further identification by mass spectrometry (MS). Spots located near the gel borders and small or faint spots were excluded from protein identification. *M. smegmatis* cells carrying pMind were used as the control in this study. The experiment was performed in triplicate.

### 2.9 Trypsin digestion, MS/MS analysis and data acquisition

The selected protein spots were excised manually from the gels for MS analysis. The subsequent trypsin digestion and MS/MS analysis were performed as described previously (Yang et al., 2018). Proteomic data were acquired using Mascot 2.2 software. The obtained MS/MS spectra were searched against the NCBI database. Confidence in the peptide identifications was assessed based on the Mascot sequence assignment score and visual inspection of the molecular mass and pI values of the selected spots from the gels.

### 2.10 Bioinformatic analysis

Gene orthology analysis was performed for differentially expressed proteins identified by MS/MS. The 18 gene symbols in *M. tuberculosis,* which were the corresponding homologues of the identified proteins in *M. smegmatis,* were input to the PANTHER database (http://www.pantherdb.org/) for functional classification. The 20 gene symbols in *M. smegmatis* were input to STRING (https://string-db.org/) for protein network construction.

## 3 RESULTS

### 3.1 Anti‐DdlA antibody specificity testing and titer determination

**Figure 1:**
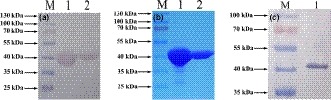
Identification of the purified DdlA by SDS‐PAGE (a) and WB (b) and specificity testing of the anti‐DdlA polyclonal antibody by WB (c). M, PageRuler prestained protein ladder (Fermentas). Colorimetric visualization was performed using BCIP/NBT solution in the WB analysis (a and c) and Coomassie in the SDS‐PAGE analysis (b). The monoclonal anti‐polyhistidine antibody at a 1:5,000 dilution was used to probe the expressed DdlA (a); the produced polyclonal anti‐DdlA antibody at a 1:2,000 dilution was used to probe the purified DdlA (c). a and b, 1‐2, the fractions of the purified DdlA. c, 1, purified *Mycobacterium smegmatis* DdlA

The amino acid sequence of *M. smegmatis* DdlA was obtained from the internet resource http://www.genome.jp/kegg/genes.html. DdlA consists of 373 aa with a molecular weight of 39.2 kDa. *M. smegmatis ddlA* was cloned into pCold II, allowing the expression of a DdlA fusion protein with a histidine tag at the N terminus and an expected molecular weight of approximately 40.2 kDa. The expressed protein was purified by Ni^2+^ affinity chromatography and examined using SDS‐PAGE and WB. Only one band was visible on the SDS‐PAGE gel and nitrocellulose membrane at 40 kDa, which agreed with the predicted molecular weight of the DdlA fusion protein (Figure 1a,b). The SDS‐PAGE and WB analyses confirmed that a soluble DdlA fusion protein had been obtained.

Female BALB/c mice were immunized with the pure recombinant DdlA antigen and sacrificed to obtain serum for testing the specificity of the anti‐DdlA antibody. Figure 1c shows that the produced anti‐DdlA antibody specifically interacted with the recombinant DdlA protein antigen. In addition, the optimum titer of 1:2,000 for the produced antibody was determined using ELISA. Therefore, the anti‐DdlA antibodies produced in this study can respond specifically against the DdlA antigen and are applicable for DdlA detection.

### 3.2 Confirmation of *M. smegmatis* cell growth inhibition and determination of decreased endogenous DdlA expression

**Figure 2:**
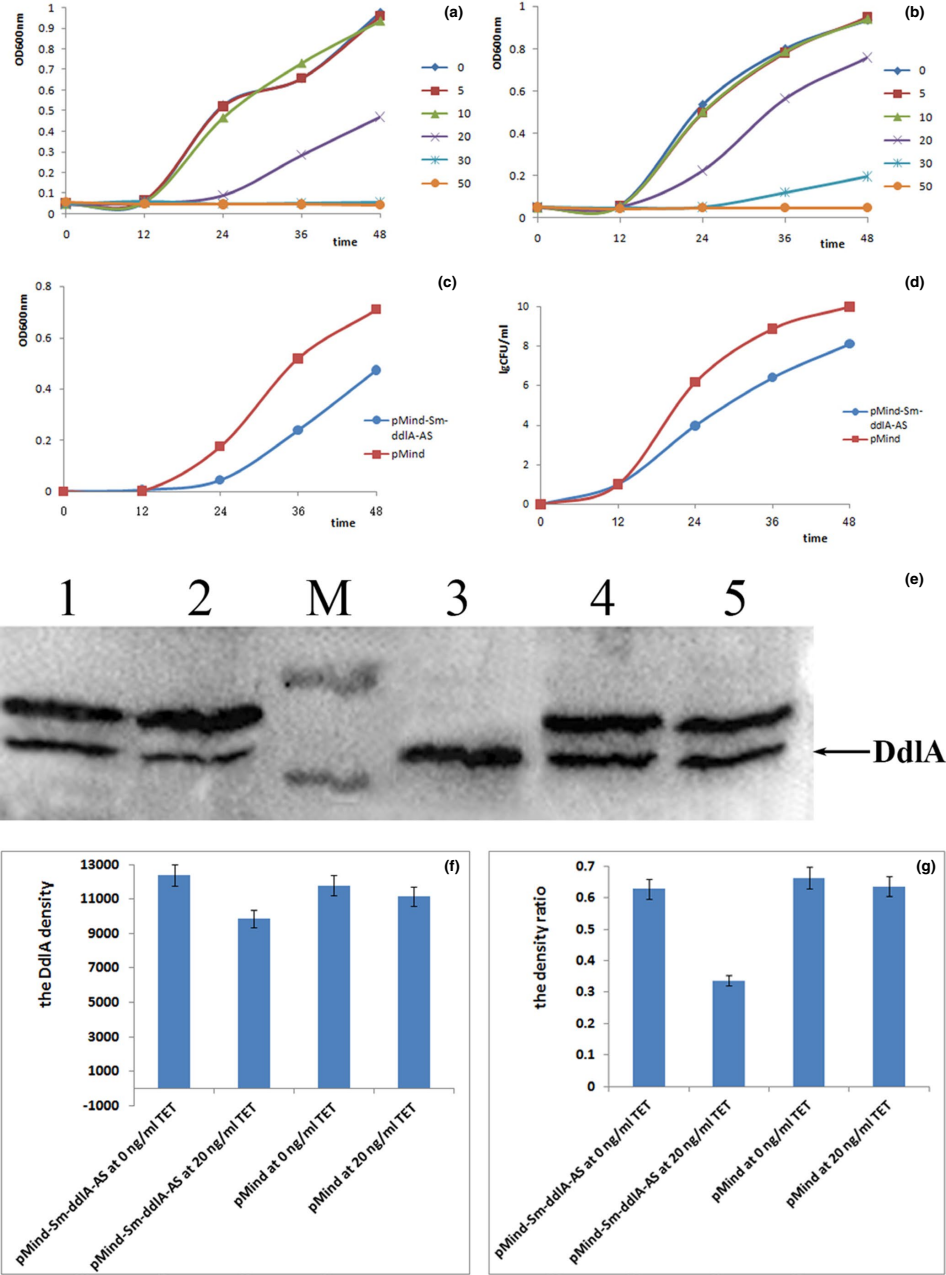
Confirmatory analysis of inhibited cell growth and decreased endogenous DdlA expression in vivo. Cell growth was monitored at 12‐hr intervals, and the values plotted are the means and standard deviations of triplicate experiments. (a) Growth curve of *Mycobacterium smegmatis* cells carrying pMind‐Sm‐ddlA‐AS grown with tetracycline concentrations of 0 ng/ml, 5 ng/ml, 10 ng/ml, 20 ng/ml, 30 ng/ml and 50 ng/ml; (b) Growth curve of *M. smegmatis* cells carrying pMind grown with tetracycline concentrations of 0 ng/ml, 5 ng/ml, 10 ng/ml, 20 ng/ml, 30 ng/ml, and 50 ng/ml; (c) Growth curve of *M. smegmatis* cells carrying pMind‐Sm‐ddlAAS and pMind grown with 20 ng/ml tetracycline; (d) CFU analysis of *M. smegmatis* cells carrying pMind‐SmddlA‐AS and pMind separately grown with 20 ng/ml tetracycline. (e) Detection of endogenous DdlA in *M. smegmatis* cells. Total protein was extracted from cells grown in the absence or presence of 20 ng/ml tetracycline for 36 h. DdlA was probed with the produced anti‐DdlA polyclonal antibody, and visualized by ECL. M, PageRuler prestained protein ladder wirh the upper and lower bands at 55 kDa and 40 kDa, respectively; 1, *M. smegmatis* cells carrying pMind‐Sm‐ddlA‐AS grown with 0 ng/ml tetracycline. 2, *M. smegmatis* cells carrying pMind‐Sm‐ddlA‐AS grown with 20 ng/ml tetracycline. 3, purified *M. smegmatis* DdlA. 4, *M. smegmatis* cells carrying pMind grown with 0 ng/ml tetracycline; 5, *M. smegmatis* cells carrying pMind grown with 20 ng/ml tetracycline. (f and g) Statistical analysis on endogenous DdlA expression in vivo. TET, tetracycline. (f) Statistical analysis of DdlA band density. (g) Statistical analysis of the density ratio of DdlA to its upper band

Tetracycline‐induced DdlA downregulation was performed in *M. smegmatis* cells carrying pMind‐*Sm‐ddlA*‐AS. *M. smegmatis* cells carrying pMind were used as the control. The growth curves of the *M. smegmatis* cells were plotted as the OD at 600 nm values versus the incubation time (Figure 2a–c). A dose‐dependent growth curve of *M. smegmatis* cells was established by adding different concentrations of tetracycline. The growth curves showed that 50 ng/ml tetracycline completely inhibited *M. smegmatis* growth, although the literature documented the inhibitory concentration of tetracycline as greater than 80 ng/ml in mycobacteria (Blokpoel et al., 2005). Tetracycline concentrations of less than 10 ng/ml did not appreciably inhibit *M. smegmatis* cell growth. However, the optimal growth inhibition occurred at final tetracycline concentrations of 30 ng/ml and 20 ng/ml. Thus, in this study, 20 ng/ml tetracycline was adopted to knock down DdlA expression (Figure 2c). The CFU plot of *M. smegmatis* cells carrying pMind‐*Sm‐ddlA*‐AS demonstrated that the quantity of surviving cells was consistent with the growth curves when 20 ng/ml tetracycline was used as an inducer. In addition, the growth curve and CFU value revealed that the optimal inhibition occurred at 36 hr.

Confirmatory studies of endogenous DdlA downregulation in *M. smegmatis* were performed by WB analysis, and the produced anti‐DdlA polyclonal primary antibody was used to probe endogenous *M. smegmatis* DdlA. Eighty micrograms of total protein from the soluble cell lysate was used for WB analysis. Figure 2e shows that two bands were visualized on the NC membrane; the lower band was identified as DdlA. The density of the lower band indicated a clear decrease in DdlA expression in the *M. smegmatis* cells carrying pMind‐*Sm‐ddlA*‐AS relative to that in the control cells (Figure 2f). In order to evaluate the expression difference of endogenous DdlA in vivo, the DdlA band density and the density ratio of DdlA band to its upper band were analyzed (Figure 2f,g). A reduction was evident for the *M.smegmatis* cells carrying pMind‐*Sm‐ddlA*‐AS grown with 20 ng/ml tetracycline compared to the control stains (the model cells: around 9,000; the control cells: more than 11,000). Additionally, the density ratio of DdlA to its upper band in the *M.smegmatis* cells carrying pMind‐*Sm‐ddlA*‐AS grown with 20 ng/ml tetracycline dramatically reduced to 33% compared to the control cells (the ratios maintained constant at around 63%). WB analysis confirmed that the expression of endogenous DdlA was significantly decreased in the *M. smegmatis* cells carrying pMind‐*Sm‐ddlA*‐AS induced by tetracycline at 20 ng/ml and was downregulated by *Sm‐ddlA* antisense RNA. Therefore, the results demonstrated that the *M. smegmatis* cell model with downregulation of DdlA by *Sm‐ddlA* antisense RNA had been successfully constructed.

### 3.3 Morphological observation revealed increased numbers of Z rings and a rearrangement of intracellular content in *M. smegmatis* cells carrying pMind‐*Sm‐ddlA*‐AS

**Figure 3:**
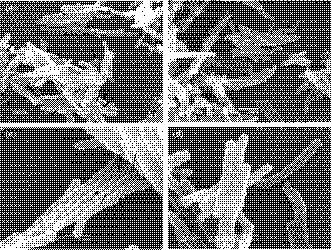
Representative SEM micrographs of *Mycobacterium smegmatis* cells. Cells were grown with 20 ng/ml tetracycline. The Z rings are indicated by arrows. (a) *M. smegmatis* cells carrying pMind‐Sm‐ddlA‐AS (magnification 8,000×); (b) *M. smegmatis* cells carrying pMind (magnification of 8,000×); (c) *M. smegmatis* cells carrying pMind‐Sm‐ddlA‐AS (magnification 20,000×); (d) *M. smegmatis* cells carrying pMind (magnification 20,000×)

SEM and TEM were used to investigate *M. smegmatis* cells carrying either pMind‐*Sm‐ddlA*‐AS or pMind at 20 ng/ml tetracycline. The SEM images (Figure 3) showed that the control cells usually appeared as straight, thin rods with a smooth cell surface and less internal specialization than the experimental cells. Like the control cells, *M. smegmatis* cells carrying pMind‐*Sm‐ddlA*‐AS appeared to be straight and rod‐like, with a smooth cell surface; however, the number of Z rings, a typical marker of cell division, was dramatically increased.

**Figure 4:**
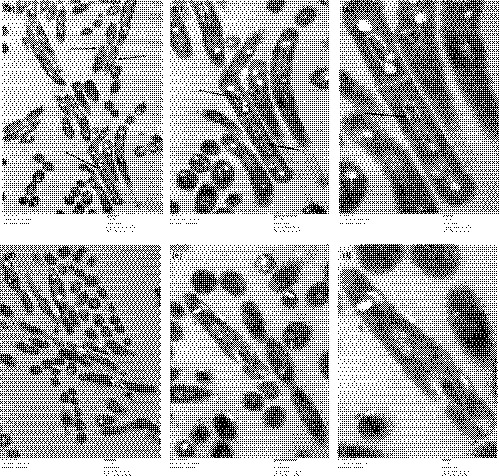
Representative TEM micrographs of *Mycobacterium smegmatis* cells. Cells were grown with 20 ng/ml tetracycline. The compact nucleoid regions and filamentous DNA are indicated by arrows in b and c; the Z rings are indicated by arrows in a. (a) *M. smegmatis* cells carrying pMind‐Sm‐ddlA‐AS (magnification 20,000×); (b) *M. smegmatis* cells carrying pMind‐Sm‐ddlA‐AS (magnification 40,000×); (c) *M. smegmatis* cells carrying pMind‐Sm‐ddlA‐AS (magnification 80,000×); (d) *M. smegmatis* cells carrying pMind (magnification 20,000×); (e) *M. smegmatis* cells carrying pMind (magnification of 40,000×); (f) *M. smegmatis* cells carrying pMind (magnification of 80,000×)

The TEM image (Figure 4a) clearly shows increased numbers of Z rings in *M. smegmatis* cells carrying pMind‐*Sm‐ddlA*‐AS, in which DdlA was downregulated by 20 ng/ml tetracycline. In addition, a clear nucleoid and filamentous DNA were visible within *M. smegmatis* cells when DdlA was downregulated by *Sm‐ddlA* antisense RNA. Therefore, the TEM images indicate that a rearrangement of intracellular content and an increase in the number of Z rings numbers occur in mycobacteria due to DdlA deficiency.

### 3.4 Identification of differentially expressed proteins by 2D‐DIGE coupled with MS/MS analysis

**Figure 5:**
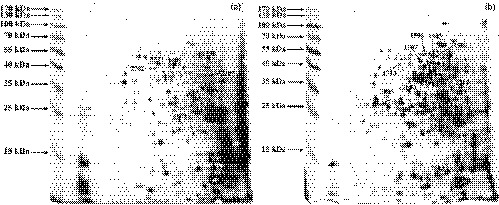
Representative maps of differentially expressed proteins as identified by 2D‐DIGE analysis. The gels were stained by silver staining. Fifty micrograms of total protein processed by the clean‐up kit was subjected to WB analysis. The numbers were labeled manually according to the sample spot (SSP) number from the PDQuest analysis. (a) *M. smegmatis* cells carrying pMind‐Sm‐ddlA‐AS grown with 20 ng/ml tetracycline for 36 hr; (b) *M. smegmatis* cells carrying pMind grown with 20 ng/ml tetracycline for 36 hr. The experiment was performed in triplicate

Proteomic changes were automatically analyzed by PDQuest software. Figure 5 shows the paired 2D‐DIGE images, which were labeled manually with the numbers obtained from the PDQuest analysis. As a consequence, 53 filtered protein spots with fold changes in expression of >2 or <0.05 displayed significant changes between the paired groups (*p* < 0.05). Of the 53 protein spots, 12 were differentially upregulated, whereas 41 were differentially downregulated in the *M. smegmatis* cell model. Considering the spot intensities and locations on the 2D‐DIGE images, 22 spots, which corresponded to the most highly differentially expressed proteins in terms of fold change and/or statistical significance, were chosen for identification by MS/MS.

Twenty‐two spots with the remarkable intensity changes were further extracted from the gel and identified by MS. Table 2 shows the properties of 20 protein spots identified by MS/MS analysis; two protein spots were excluded because they could not be identified by MS. Of the 20 candidates, six proteins, namely, mitomycin antibiotic/polyketide fumonisin biosynthesis protein, the elongation factor Tu, a bifunctional o‐acetylhomoserine, an M18 family aminopeptidase, a putative acetamidase/formamidase, and NAD(P)H‐quinone dehydrogenase, were significantly upregulated in the *M. smegmatis* cells carrying pMind‐*Sm‐ddlA*‐AS. In contrast, 14 proteins, namely, SAM‐dependent methyltransferase, the heat shock protein hspX, the acetyl‐CoA acetyltransferase Fada6 (acetoacetyl‐CoA thiolase), the carbohydrate kinase CbhK, an aldehyde dehydrogenase (NAD) family protein, aspartyl/glutamyl‐tRNA amidotransferase subunit A, cystathionine beta‐synthase, the phage shock protein PspA, N‐nitrosodimethylamine (NDMA)‐dependent methanol dehydrogenase, a phosphoglycerate kinase, NAD(P)H nitroreductase, class II fumarate hydratase, enoyl‐ACP reductase, and acetyl‐CoA acetyltransferase, were significantly downregulated in *M. smegmatis* cells carrying pMind‐*Sm‐ddlA*‐AS relative to their expression in *M. smegmatis* cells carrying pMind.

**Table 2:** Properties of the 20 differentially expressed proteins identified by MS

Protein Spot No. on the 2D Gel	ddlA/pMind	Protein Name	NCBI Accession No.	No. in *Mycobacterium smegmatis*	No. in *Mycobacterium tuberculosis*	MW (Da)	PI	Matched Peptide Count	Score	Protein Score CI (%)
5403	0.42	SAM‐dependent methyltransferase	WP_011731145.1	MSMEG_6235	Rv3699	24485.2	5.01	7	278	100
7403	0.05	Heat shock protein hspX	CKI07130.1	MSMEG_3932	Rv2031c	15917.1	4.93	9	307	100
2506	0.26	Acetyl‐CoA acetyltransferase Fada6 (acetoacetyl‐CoA thiolase)	CKI42884.1	MSMEG_6008	Rv3556c	40639.6	5.01	16	157	100
2217	0.11	Carbohydrate kinase CbhK	CKI12759.1	MSMEG_4270	Rv2202c	34832.4	4.62	16	208	100
1503	0.28	Aldehyde dehydrogenase (NAD) family protein	ABK71967.1	MSMEG_1665	Rv0768	51884.1	4.9	16	169	100
1509	0.19	Aspartyl/glutamyl‐tRNA amidotransferase subunit A	WP_003893733.1	MSMEG_2365	Rv3011c	51240.8	4.99	16	322	100
1504	0.28	Cystathionine beta‐synthase	CKI28531.1	MSMEG_5270	Rv1077	48750.9	5.03	12	281	100
3805	0.33	Phage shock protein PspA	CKH69159.1	MSMEG_2695	Rv2744c	30273.2	5.55	16	231	100
1713	0.18	NDMA‐dependent methanol dehydrogenase	WP_011731151.1	MSMEG_6242	Rv1530	46262	5.36	21	607	100
2305	0.35	Phosphoglycerate kinase	YP_887401.1	MSMEG_3085	Rv1437	42068.9	4.67	21	896	100
3603	0.05	NAD(P)H nitroreductase	AIU23528.1	MSMEG_5246	Rv2032	36198.5	5.19	20	581	100
1607	0.23	Class II fumarate hydratase	WP_011730408.1	MSMEG_5240	Rv1098c	49731.8	5.15	14	129	100
4710	0.46	Enoyl‐ACP reductase	YP_887466.1	MSMEG_3151	Rv1484	28508.8	5.28	13	406	100
2504	0.21	Acetyl‐CoA acetyltransferase	AIU23553.1	MSMEG_5273	Rv1074c	42564.3	4.96	25	743	100
5501	57.69	Mitomycin antibiotics/polyketide fumonisin biosynthesis protein	CKH22930.1	MSMEG_1419	NA	25674	5.02	10	234	100
1611	18.82	Elongation factor Tu	YP_885786.1	MSMEG_1401	Rv0685	43708.6	5.18	21	1,030	100
1609	5.61	Bifunctional o‐acetylhomoserine	WP_003893059.1	MSMEG_1652	Rv3340	46795	5.12	13	648	100
2702	3.21	M18 family aminopeptidase	WP_011730828.1	MSMEG_5828	Rv0800	44851.4	5.25	18	196	100
1350	3.95	Putative acetamidase/formamidase	CKI14571.1	MSMEG_4367	NA	44469.1	4.67	13	152	100
1709	3.16	NAD(P)H‐quinone dehydrogenase	WP_011727859.1	MSMEG_1735	Rv3303c	49036.8	5.37	18	86	99.983

ddlA/pMind > 1, upregulation; ddlA/pMind < 1, downregulation.

## 4 DISCUSSION

To gain insights into the influence of DdlA on mycobacterial morphology, an ultrastructural analysis was carried out by TEM and SEM. The electron micrograph of *M. smegmatis* cells grown under DdlA deficiency shows an increase in the number of Z rings and a rearrangement of the intracellular contents, with a clear nucleoid and filamentous DNA, whereas control cells exhibited a regular length and a very homogeneous electron density. In light of the growth inhibition, we hypothesized that the parent *M. smegmatis* cells carrying the newly synthesized daughter DNA could not divide into two daughter cells due to the DdlA deficiency. Therefore, DdlA deficiency attenuated mycobacterial cell division. This result was consistent with the literature describing cell morphology in cell division (Cambridge, Blinkova, Magnan, Bates, & Walker, 2014; Marteyn et al., 2014; Szwedziak, Wang, Bharat, Tsim, & Jan, 2014). However, the mechanism by which DdlA influences mycobacterial division remains to be determined. Additionally, the morphological images showed that although DdlA deficiency could not directly lead to mycobacterial death, it efficiently inhibited mycobacterial growth by attenuating cell division. Therefore, drugs targeting DdlA are still attractive for the treatment of TB. Thus, the combination of compounds targeting DdlA with first‐line anti‐TB drugs such as isoniazid, rifampicin, ethambutol, pyrazinamide, and streptomycin, may be more effective as TB medications due to their different inhibition mechanisms.

**Figure 6:**
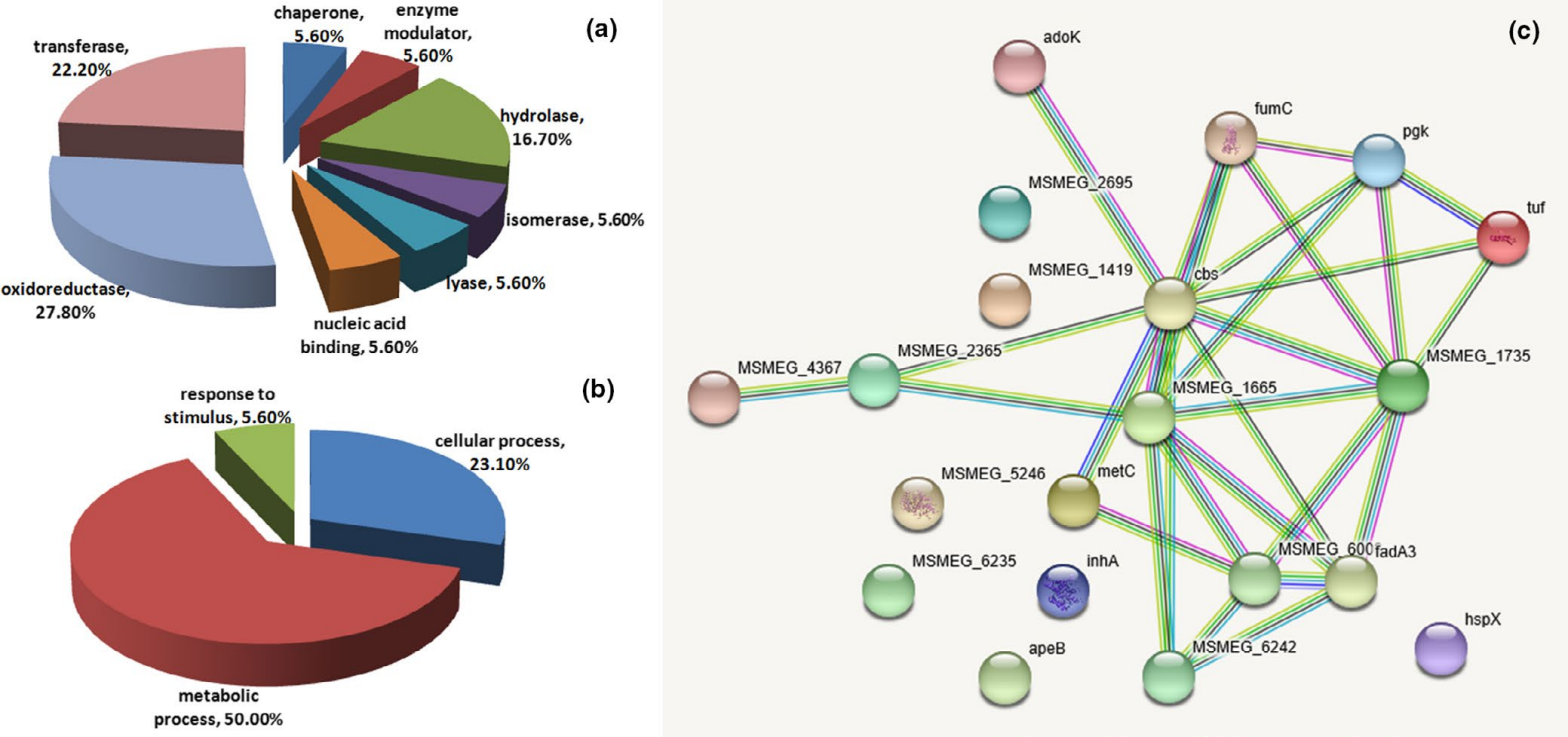
Bioinformatic analysis of 20 differentially expressed proteins identified by MS/MS between the control and DdlA downregulation by ddlA antisense RNA groups. (a and b) Protein classification analysis using the PANTHER database. The representative pie chart displays the enrichment percentage for different functional categories of proteins. (a) Proteins were categorized into groups according to protein class; (b) Proteins were categorized into groups according to biological process; (c) Protein‐protein interaction network analysis using the STRING database

To reveal the mechanism of action and relevant regulatory effects of DdlA on cell growth under DdlA deficiency, proteomic analysis of soluble proteins from *M. smegmatis* cells with DdlA downregulation was carried out by 2D‐DIGE coupled with MS/MS analysis. In this study, 20 proteins were identified to be significantly differentially regulated. Of the 20 identified proteins, the most highly differentially expressed proteins were involved in metabolic processes (Figure 6b) and were in the class of oxidoreductases (Figure 6a). Based on metabolic processes involving oxidoreductases, we examined the possible variance in the metabolism of different nutrients, including carbohydrates, lipids, and proteins. We found that the proteins involved in carbohydrate catabolism (MSMEG_4270, MSMEG_3085, and MSMEG_5240) were downregulated and that the proteins involved in the biosynthesis of fatty acids (MSMEG_6008, MSMEG_3151, and MSMEG_5273) were also downregulated. However, the enzymes promoting protein degradation (MSMEG_5828) and synthesis (MSMEG_1401) were upregulated. Thus, we proposed that DdlA deficiency led to a reduction in carbohydrate catabolism and an inhibition of fatty acid anabolism. Additionally, both degradation and synthesis of proteins remained in an active state under DdlA deficiency.

Interestingly, NDMA‐dependent methanol dehydrogenase (MSMEG_6242) was identified as a differentially expressed protein under DdlA deficiency; it was also identified as a differentially expressed protein when *M. smegmatis* growth was inhibited by the taurine‐5‐bromosalicylaldehyde Schiff base compound (data not published). NDMA‐dependent methanol dehydrogenase allows gram‐negative microorganisms to generate energy from methanol oxidation and to synthesize compounds with carbon–carbon bonds from methanol assimilation (Harm, Harm, & Dijkhuizen, 2000). In this study, MSMEG_6242 was found to directly interact with MSMEG_1735, MSMEG_6008, MSMEG_1665, and MSMEG_5273 to form a network regulating *M. smegmatis* growth (Figure 4c). Thus, MSMEG_6242 may play an important role in regulating *M. smegmatis* growth under adverse environmental conditions and could be developed into an anti‐TB drug target. Additionally, although the use of carbohydrates as carbon sources was limited due to the low activity of MSMEG_6242, acetamidase (MSMEG_4367), an inducible enzyme, was activated under DdlA deficiency. This enzyme enables *M. smegmatis* to utilize several amides as carbon sources.

In this study, the most highly differentially expressed protein was MSMEG_1419, which was highly upregulated when *M. smegmatis* was grown under DdlA deficiency. MSMEG_1419 encodes fumonisin, which usually exists in contaminated maize (Brown, Butchko, Busman, & Proctor, 2007). Fumonisin, a notorious mycotoxin, consists of aminopolyols with a core structure containing a 19‐ or 20‐carbon backbone with methyl, hydroxyl, and tricarballylic acid moieties at different sites along the carbon backbone (Baird et al., 2008). Currently, accumulating evidence shows that fumonisin, which is a polyketide derivative and is structurally related to sphinganine, usually causes a variety of toxicological effects, such as esophageal cancer, cardiovascular problems, and neural tube defects, in humans and animals (Desjardins, Munkvold, Plattner, & Proctor, 2002; Waskiewicz, Stepien, Wilman, & Kachlicki, 2013). Currently, little is known about the roles of fumonisin (MSMEG_1419) in mycobacteria and its homologous protein in *M. tuberculosis*. However, such evidence may expand our approaches toward antimycobacterial drug design for targeting highly upregulated mycotoxin.

Since the first report of the introduction of foreign DNA into *M. smegmatis* in 1987, *M. smegmatis* has been used as the workhorse of mycobacterial research and as a surrogate model for pathogenic and slower‐growing mycobacterial species such as *M. leprae* and *M. tuberculosis* (Liu, Yang, & He, 2016; Titgemeyer et al., 2007). In this study, *M. smegmatis* was utilized as a surrogate host for *M. tuberculosis* to further investigate the roles of DdlA in mycobacteria. Additionally, sequence alignment of *Tb*‐DdlA and *Sm*‐DdlA showed that *Sm*‐DdlA was highly homologous to *Tb*‐DdlA (87% amino acid sequence identity). Therefore, *M. smegmatis* was applicable for demonstrating the influence of DdlA on the survival, morphology and proteome of mycobacteria. Despite the limitations inherent to the use of *M. smegmatis,* the findings of this study may provide valuable insight into anti‐TB drug design to combat the difficult‐to‐treat pathogen *M. tuberculosis*.

## 5 CONCLUSIONS

In summary, we successfully constructed the *M. smegmatis* cell model with DdlA downregulation by *Sm‐ddlA* antisense RNA. Based on this model, we found that DdlA deficiency stimulated the generation of an increased number of Z rings and the rearrangement of intracellular contents with a clear nucleoid and filamentous DNA, by *M. smegmatis*, which further inhibited mycobacterial cell division. Proteomic analysis identified 20 differentially expressed proteins, including six that were significantly upregulated and 14 that were significantly downregulated. Bioinformatic analysis showed that DdlA deficiency attenuated the process of carbohydrate catabolism and the pathway of fatty acid anabolism, while maintaining active protein degradation and synthesis. In addition, NDMA‐dependent methanol dehydrogenase (MSMEG_6242) and fumonisin (MSMEG_1419) were the identified proteins with the greatest differential expression; these proteins have substantial potential to be developed into anti‐TB drug targets. Therefore, our dataset provides intriguing insight into antimycobacterial drug design via developing novel anti‐TB drug targets which were revealed in this study.

## CONFLICT OF INTERESTS

The authors declared no conflict of interest.

## AUTHORS CONTRIBUTION

W.Z. conducted experiments and modified the manuscript. Y.C. composed and modified the manuscript. Y.X., W.D., and S.Y. contributed to funding support. S.L. processed and analyzed the data. All authors shared in responsibility for the final decision to submit it for publication.

## ETHICS STATEMENT

None required.

## Data Availability

All data are provided in full in the results section of this paper.
